# Acute toxicity of the insecticide Imidacloprid and the herbicide 2,4-D in two species of tropical anurans

**DOI:** 10.1007/s10646-024-02843-y

**Published:** 2025-02-01

**Authors:** Teófila María Triana Velásquez, Manuel Hernando Bernal Bautista

**Affiliations:** Grupo de Herpetología, Eco-Fisiología & Etología, Departamento de Biología, Universidad del Tolima, Ibagué, Colombia

**Keywords:** Amphibians, LC_50_, Pesticides, Sublethal effects, Toxicity

## Abstract

The use of pesticides has notably increased in recent years globally. However, sensitive organisms exposed to these environmental pollutants, such as amphibians, may experience adverse effects. The insecticide imidacloprid (IM) and the herbicide 2,4-dichlorophenoxyacetic acid (2,4-D) are two pesticides commonly used in Colombia, but their toxic impacts on tropical anurans remain poorly understood. In this study, we tested the acute toxic effects of IM and 2,4-D on the survival, total length, and burst swimming speed of tadpoles from two anuran species. Under laboratory conditions, the tadpoles of *Boana platanera* and *Engystomops pustulosus* were independently exposed to each pesticide for 96 h. We found that the tadpoles of *E. pustulosus* were more sensitive to both IM and 2,4-D than those of *B. platanera*. However, the LC_50_ values were higher than the reported field concentrations for these pesticides. IM led to a reduction in the total length of *B. platanera* tadpoles and induced total immobility in surviving individuals of both species. In contrast, the herbicide 2,4-D did not affect the total length or the swimming speed of tadpoles from the two species. In conclusion, based on the results and the reported field concentrations, IM and 2,4-D are not lethal to the studied anurans. Nevertheless, it is important to consider that IM caused strong negative sublethal effects on tadpoles, which could compromise their survival in the future. Finally, we also found that the insecticide IM showed notably greater toxicity to the tested species than did the herbicide 2,4-D.

## Introduction

Colombia is an agricultural country highly dependent on the intensive application of pesticides, such as fungicides, insecticides, and herbicides (ICA 2021). Imidacloprid (IM), (1-(6-chloro-3-pyridylmethyl)-N-nitroimidazolidin-2-ylideneamine), is an agrochemical belonging to a class of insecticides called neonicotinoids. Neonicotinoid insecticides are widely used around the world, principally in many countries of South America, although most of the outdoor uses of neonicotinoids are banned by the European Union (Fonseca et al. 2022). The IM acts on postsynaptic nicotinic acetylcholine (https://www.sciencedirect.com/topics/earth-and-planetary-sciences/acetylcholine) receptors in the central nervous system, inducing spontaneous discharge, after which the neuron is unable to propagate further impulses, and insects die (Nunges et al. 2023). IM has been classified as moderately hazardous (Class II) by the World Health Organization (WHO) (1990). IM is primarily intended for insect pest management and is considered safe for vertebrates due to its low affinity for nicotinic receptors (Dalefield 2017).

IM has a half-life of 39 days at the soil surface and a high-water solubility (0.51 g L^−1^ at 20 °C) (Fulton et al. 2013), which makes it an efficient systemic agrochemical but also highly susceptible to leaching and runoff into ground and surface waters (Anderson et al. 2015; Morrissey et al. 2015). Several studies have recently reported the presence of neonicotinoid insecticides in natural waters worldwide (Anderson et al. 2015; Morrissey et al. 2015; López-Pacheco et al. 2019) and have assessed their toxicity in aquatic vertebrates. Based on available data, acute toxicity values for aquatic vertebrates, amphibians, and fishes indicate a low risk of direct mortality, as the median lethal concentration values (LC_50_) are in the parts per million range (Lewis et al. 2016; Fonseca et al. 2022; National Center for Biotechnology Information - NCBI 2023a), which is much greater than the concentrations reported in the environment (Morrissey et al. 2015).

On the other hand, the herbicide 2,4-dichlorophenoxyacetic acid (2,4-D) has been used as a pesticide since the 1940s to control broadleaf weeds in both agricultural and nonagricultural plantations, encompassing terrestrial and aquatic environments (Dehnert et al. 2021). 2,4-D has been classified as moderately hazardous (Class II) by the WHO (1990) and as possibly carcinogenic to humans (Group 2B) by the IARC (2015). The half-life of 2,4-D in aerobic aquatic environments is estimated to be 10–>50 days (NCBI 2023b). In addition, it has a low adsorption coefficient and high solubility in water (600 mg L^−1^ at 25 °C) (Islam et al. 2018). Previous toxicity studies of 2,4-D have revealed that this herbicide causes multiple physiological, morphological, histological, and genotoxic alterations in the embryos and larvae of the fishes *Danio rerio* (Li et al. 2017) and *Cnesterodon decemmaculatus* (De Arcaute et al. 2016), and the amphibians *Xenopus laevis* (LaChapelle et al. 2007; Stebbins‐Boaz et al. 2004), *Physalaemus albonotatus* (Curi et al. 2019), *Lithobates catesbeianus*, *Leptodactylus fuscus*, *Physalaemus nattereri* (Freitas et al. 2019), and *Rhinella arenarum* (Aronzon et al. 2011). Nevertheless, many of these studies have shown that 2,4-D has toxic effects on aquatic vertebrates at concentrations exceeding those typically found in natural environments (e.g., Figueiredo et al. 2014; De Arcaute et al. 2016; Li et al. 2017).

Amphibians currently represent the most threatened group of vertebrates, with one-third of known species at risk of extinction (IUCN 2024), while others are facing rapid population declines (Campbell et al. 2020; Green et al. 2020; Lötters et al. 2023). Numerous global and local factors have been attributed to this decline (Angelini et al. 2020), but the use of agrochemicals to increase crop productivity is a notable concern because of the growing evidence of their adverse effects on the water quality of amphibian habitats. For instance, when agrochemicals are sprayed, shallow freshwater pools, which are essential habitats for many amphibian species, are frequently the most affected areas (Sparling et al. 2010). This issue is especially critical for the early aquatic developmental stages of amphibians (embryos, larvae, tadpoles), which are very sensitive to environmental contaminants due to their permeable skin and limited ability to disperse (Greulich & Pflugmacher 2003; Yan et al. 2008; Orizaola 2022). Consequently, pesticide application can have an important impact on amphibian populations.

Colombia has the second-highest amphibian diversity worldwide, with 859 species, surpassed only by Brazil, with 1233 species (Frost 2023). Moreover, Colombia ranks second in pesticide consumption per hectare in Latin America, which has nearly quadrupled over the past two decades (The Food and Land Use Coalition 2023). Therefore, in this study, we investigated the acute toxic effects of the insecticide IM and the herbicide 2,4-D, both pesticides widely used in the Department of Tolima, Colombia, on the survival, burst swimming speed, and total length of tadpoles from two anuran species frequently exposed to these agrochemicals. In addition, we compared the differential toxicity of IM and 2,4-D to the studied species.

## Materials and methods

### Test organisms

We evaluated the toxic effects of IM and 2,4-D on the tadpoles of two anuran species, *Boana platanera* (Escalona M et al. 2021), and *Engystomops pustulosus* (Cope 1864). We chose these species because they are common in Colombia, are in a low-risk category of extinction (IUCN 2024) and live in farmlands where their tadpoles develop in ponds that are frequently sprayed with these pesticides. Tadpoles were obtained from egg masses collected directly in a pond located in the corregimiento Potrerillo (04°15’ N, and 74°58’ W), municipality of Coello, Department of Tolima, Colombia, and from a temporary pond in the municipality of Ibagué (4°25’ N, and 75°11’ W), also in the Department of Tolima, Colombia. These ponds are pesticide-free, according to information from local residents and our fieldwork conducted in the area from approximately 10 years ago. Subsequently, the egg masses were transported to the Laboratory of Herpetology at the University of Tolima, Ibagué, Colombia, where the embryos were raised to the experimental Gosner’s stage 25 (Gosner 1960) in tanks filled with aerated tap water maintained at a temperature of 23–25 °C. Tadpoles were not provided with food either before or during the experiments to prevent problems caused by feeding, such as the possible alteration of the toxicant concentration, the build-up of food and metabolic wastes, and resulting oxygen demand (USEPA 2002). After the end of the experiments, the surviving tadpoles were placed in aerated tap water at the laboratory’s environmental temperature to raise them to metamorphosis. However, all tadpoles died later.

### Testing procedures

Commercial formulations of the insecticide IM (Imidacloprid 350 SC-DVA) and the herbicide 2,4-D (Fedeamina 720 SL) were obtained from agricultural retailers and stored separately at room temperature. Imidacloprid 350 SC-DVA contains 350 g L^−1^ of Imidacloprid (1-(6-chloro-3-pyridylmethyl)-N-nitroimidazolidin-2-ylideneamine) as the active ingredient, whereas Fedeamina 720 SL contains 720 g L^−1^ of acid 2,4-D, equivalent to 867.5 g L^−1^ of the dimethyl-amine salt (DMA). The water used for both the control and treatment groups was identical to that utilized for raising the tadpoles (tap water dechlorinated by aeration). The mean physicochemical parameters of this water were: pH = 7.62 ± 0.32, temperature = 23.1 ± 0.87 °C, conductivity = 324.15 ± 49.5 μS cm^−1^, and dissolved oxygen = 6.97 ± 0.60 ppm. These values are within the acceptable range for tadpole testing (USEPA 2002).

Test chambers consisted of 2 L glass bowls containing 1 L of test solution. These chambers were positioned indiscriminately by the treatment group in an air-conditioned area of the laboratory designed to maintain a similar temperature (23 ± 2 °C) and photoperiod (12:12 h light: dark cycle) throughout the experiments. A primary stock solution was separately prepared for each pesticide (IM and 2,4-D) by dissolving the formulated IM and 2,4-D in dilution water to achieve nominal concentrations of 700 mg L^−1^ (IM) and 864 mg L^−1^ (2,4-D), respectively. Nominal test concentrations were selected based on the results of range-finding tests, as suggested by USEPA (2002). In these tests, we conducted multiple abbreviated static acute experiments, exposing 10 organisms to a broad range of geometric dilutions for 24 h initially, and then for 96 h, to identify the concentrations to use for definitive tests. The definitive tests for both pesticides consisted of four concentrations and a negative control, which provided a dose-response curve.

Three replicates of each test solution were prepared by serial dilution of the stock solution with dilution water to yield a range of nominal concentrations in 1 L of solution for each pesticide (IM: 43.7, 87.5, 175, 350 mg L^−1^; and 2,4-D: 54, 108, 216, 432 mg L^−1^). Ten tadpoles were indiscriminately placed in each of the three test chambers, for a total of 30 tadpoles per concentration. The rate of biomass loading, defined as the total wet weight of 10 tadpoles L^−1^ of test water, was kept below 0.6 g L^−1^, as recommended in ASTM guidelines (ASTM 1998). The test solutions were renewed daily by transferring the tadpoles to newly prepared solutions during the 96 h of the experiments.

### Measurements of mortality and sublethal effects

Mortality was determined based on the accumulated number of dead tadpoles after 96 h of exposure. The median lethal concentration (LC_50_) and the associated 95% confidence intervals (CI) were estimated using the TSK Trimmed Spearman–Karber method (version 1.5). Sublethal effects at 96 h were assessed by measuring tadpole length and burst swimming performance, a commonly used proxy for amphibian fitness (Watkins 1996). For these experiments, we tested the surviving tadpoles from the control group for IM (29 for both species) and 2,4-D (30 for both species). Additionally, we tested 30 (*B. platanera*) and 16 (*E. pustulosus)* tadpoles for the lowest concentration of IM (43.7 mg L^−1^); and either 30 or 29 (*B. platanera*) and 30 or 26 tadpoles (*E. pustulosus*) for the two lowest concentrations of 2,4-D (54, 108 mg L^−1^), respectively. Photographs of individual tadpoles were taken to measure the total length (TL: distance between the snout and tip of the tail fin) through the software ImageJ (http://rsbweb.nih.gov/ij/). This information was analyzed by ANOVA for each species.

For the burst swimming performance test, tadpoles were individually induced to swim three times in a water-filled rectangular plastic tray (10 × 1 × 1.5 cm deep) at 25 °C by applying a tactile stimulus to the tail. This tactile stimulus was applied consistently with the tip of a brush pencil on the tadpole’s tail. Tadpoles were recorded with a video recorder (Panasonic HC-V750). Afterward, the software Tracker Video Analysis and Modelling Tool (version 6.1.3) was utilized to select the maximum speed (in cm s^−1^) reached by each tadpole during the first 0.5 s among the three tests. This maximum speed was used for the statistical analysis. Each tadpole was then photographed, and its total length was measured with the software ImageJ (http://rsbweb.nih.gov/ij/). These data were evaluated by an ANCOVA using the total length as a covariate. All statistical analyses were conducted with the IBM SPSS Statistics program (version 21) (IBM Corporation 2012).

### Analytical methods

ELISA test kits manufactured by Eurofins Abraxis (Warminster, PA) were used to measure the concentrations of IM (reference: PN 500800) and 2,4-D (reference: PN 54003A) in the test solutions. Each experimental solution was diluted 1:300 in water before analysis. Samples outside the standard curves were further diluted as necessary. The point calibration curves of both pesticides were employed as proposed in the manual guide of the Eurofins Abraxis assay kit (IM: from 0.075 to 1.2 μg L^−1^; 2,4-D: from 2.0 to 8.0 μg L^−1^). Additionally, following the manual guide, the % B/Bo was reported as the mean absorbance value for each standard (B) divided by the mean absorbance value for the Diluent/Zero Standard (Bo). Then, the concentration of each experimental solution was calculated by interpolation on the standard curve and by correcting for the mean quality control (QC) percent recovery, based on analyses of two replicates of one concentration (IM: 0.075 μg L^−1^; 2,4-D: 2 μg L^−1^) of the standard. The final concentration of agrochemicals in the exposure solution was determined by multiplying the result by the dilution factor of 300, as proposed in the manual guide. The method limit of quantitation (LOQ) for this analysis was defined as the lowest calibration standard of 0.075 μg L^−1^ (for IM) and 2.0 μg L^−1^ (for 2,4-D). Three matrix blank samples were analyzed to identify possible interferences. No interferences were detected above the LOQ during the sample analyses.

## Results

### Measurement of test concentrations

#### IM

Samples collected at test initiation had measured concentrations of IM that ranged from 91 to 130% of the nominal concentrations. Samples collected prior to the renewal of the test solutions at 24 h contained measured concentrations that ranged from 79 to 105% of the nominal concentrations. When the measured concentrations of the samples collected at 0, 24, and 96 h were averaged, the mean measured concentrations ranged from 91.4 to 111.6% of the nominal concentrations. Because the measured values were close to the nominal concentrations, we used the nominal concentrations to determine the LC_50_ values and to compare our results with those from other studies.

#### 2,4-D

Samples collected at test initiation had measured concentrations of 2,4-D that ranged from 92 to 130% of the nominal concentrations. Samples collected prior to the renewal of the test solutions at 24 h contained measured concentrations that ranged from 82 to 104% of the nominal concentrations. When the measured concentrations of the samples collected at 0, 24, and 96 h were averaged, the mean measured concentrations ranged from 92.5 to 112.7% of the nominal concentrations. Because the measured values were close to the nominal concentrations, we also used the nominal concentrations to determine the LC_50_ values and to compare our results with those from other studies.

### Lethal effects

**Fig. 1:**
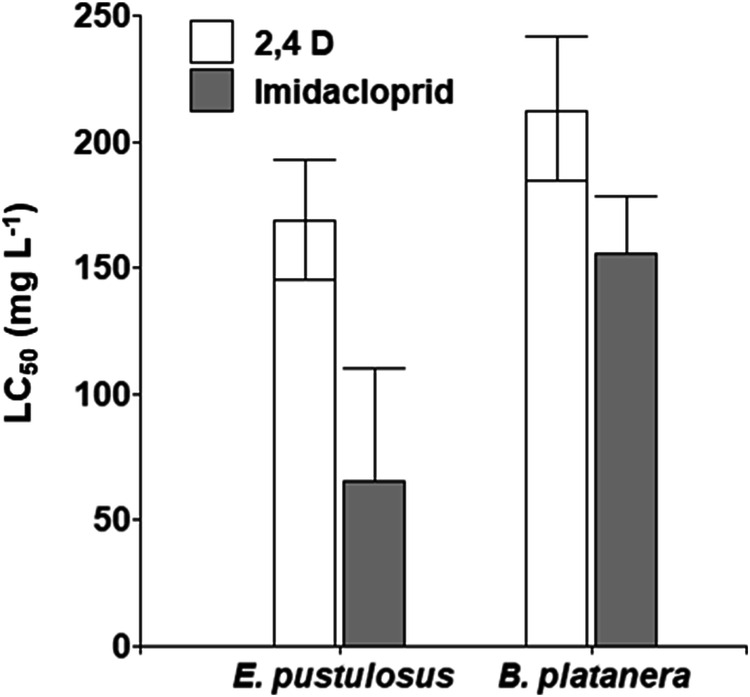
Toxicity values (LC_50_) for *B. platanera* and *E. plustulosus* tadpoles exposed for 96 h to the pesticides IM and 2,4-D. The bars represent the 95% confidence intervals

**Fig. 2:**
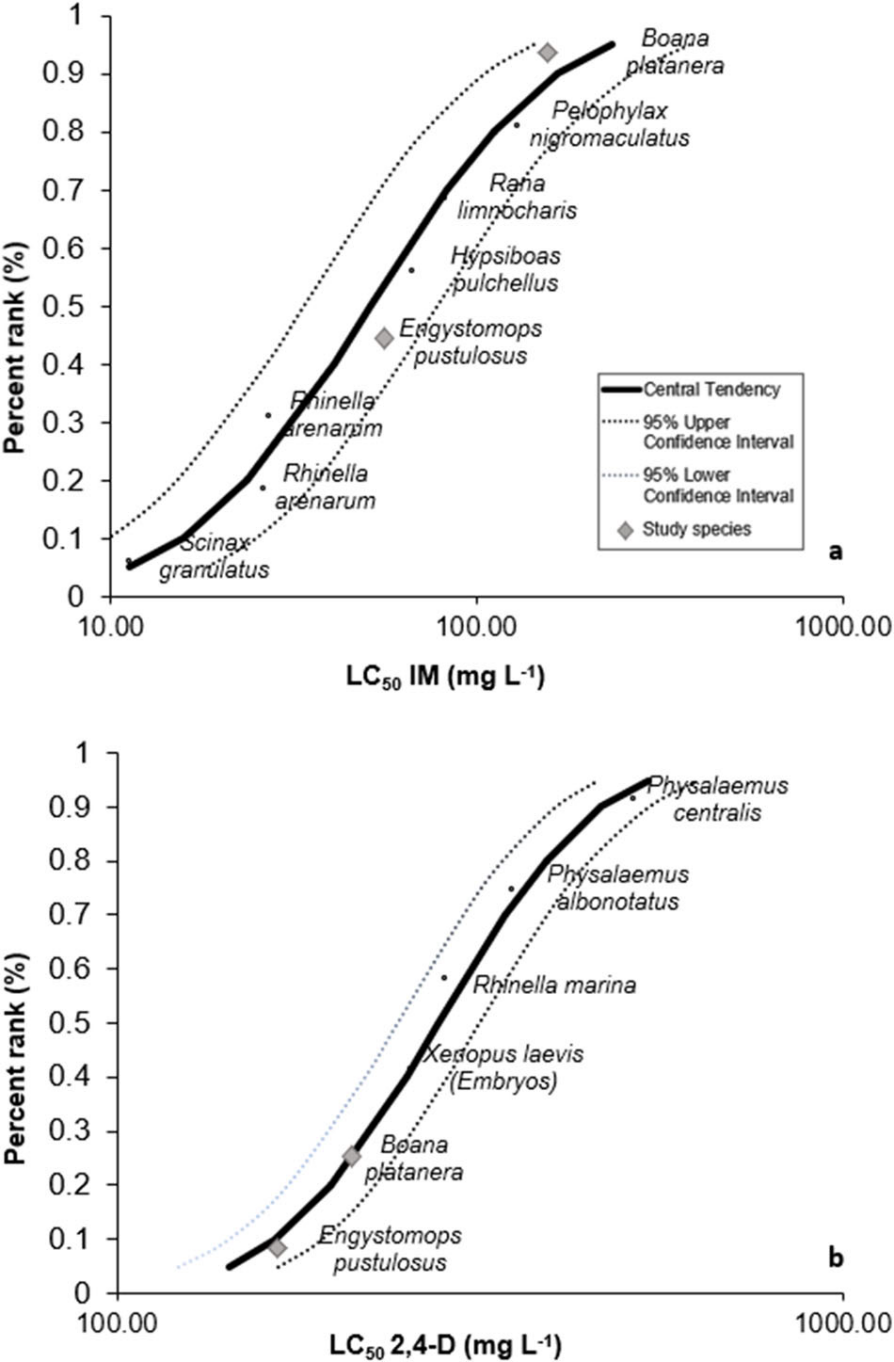
Species sensitivity distribution (SSD) of LC_50_ values for Colombian tadpoles exposed in this study to the commercial formulations of IM (**a**) or 2,4-D (**b**), as well as for other commercial formulations or active ingredients of these pesticides, as indicated in Curi et al. (2019), Feng et al. (2004), Figueredo and de Jesus Rodrigues (2014), Fonseca et al. (2022), Morgan (1996), Pérez-Iglesias et al. (2014), and De Arcaute et al. (2014)

The control treatments in all the experiments resulted in low mortality, between 0 and 3.3% (Table 1). *E. pustulosus* was more sensitive to IM (LC_50_: 56.74 mg L^−1^) and 2,4-D (LC_50_: 167.52 mg L^−1^) than was *B. platanera* (IM: LC_50:_ 154.69 mg L^−1^; 2,4-D: LC_50:_ 211.07 mg L^−1^) (Table 1, Fig. 1). The LC_50_ values were combined with other published data for the two pesticides in a species sensitivity distribution graph (SSD) (Fig. 2). The fifth centiles of the toxicity distribution were 11.317 mg L^−1^ for IM and 142.807 mg L^−1^ for 2,4-D. These data indicate that the Colombian species *E. pustulosus* and *B. platanera* fall within 95% of the toxicity range reported for IM and 2,4-D formulations. Nevertheless, according to this SSD, tadpoles of *B. platanera* are the most tolerant to IM (Fig. 2a), whereas tadpoles of both *E. pustulosus* and *B. platanera* are the most sensitive to 2,4-D (Fig. 2b). Finally, the LC_50_ values were lower for the insecticide IM than for the herbicide 2,4-D in both species studied (Fig. 1).

**Table 1:** Percent mortality, LC_50_ values, and 95% confidence intervals for the *B. platanera* and *E. plustulosus* tadpoles exposed for 96 h to the pesticides 2,4-D and IM

Herbicide 2,4-D						
Species	Concentrations (mg L^−1^)					LC_50_ (mg L^−1^)
	0	54	108	216	432	
***B. platanera***	0	0	3.33	15	100	211.07 (184.51–241.44)
***E. pustulosus***	0	0	13.33	73.33	100	167.52 (147.47–192.92)
**Insecticide IM**						
**Species**	**Concentrations (mg L**^**−1**^**)**					**LC**_**50**_ **(mg L**^**−1**^**)**
	0	43.7	87.5	175	350	
***B. platanera***	3.33	0	10	60	100	154.69 (134.09–178.46)
***E. pustulosus***	3.33	46.66	60	100	100	56.74 (29.22–110.15)

InfoStat version 2018 and Microsoft Office Excel were used to generate the figures presented in this study

### Sublethal effects

**Fig. 3:**
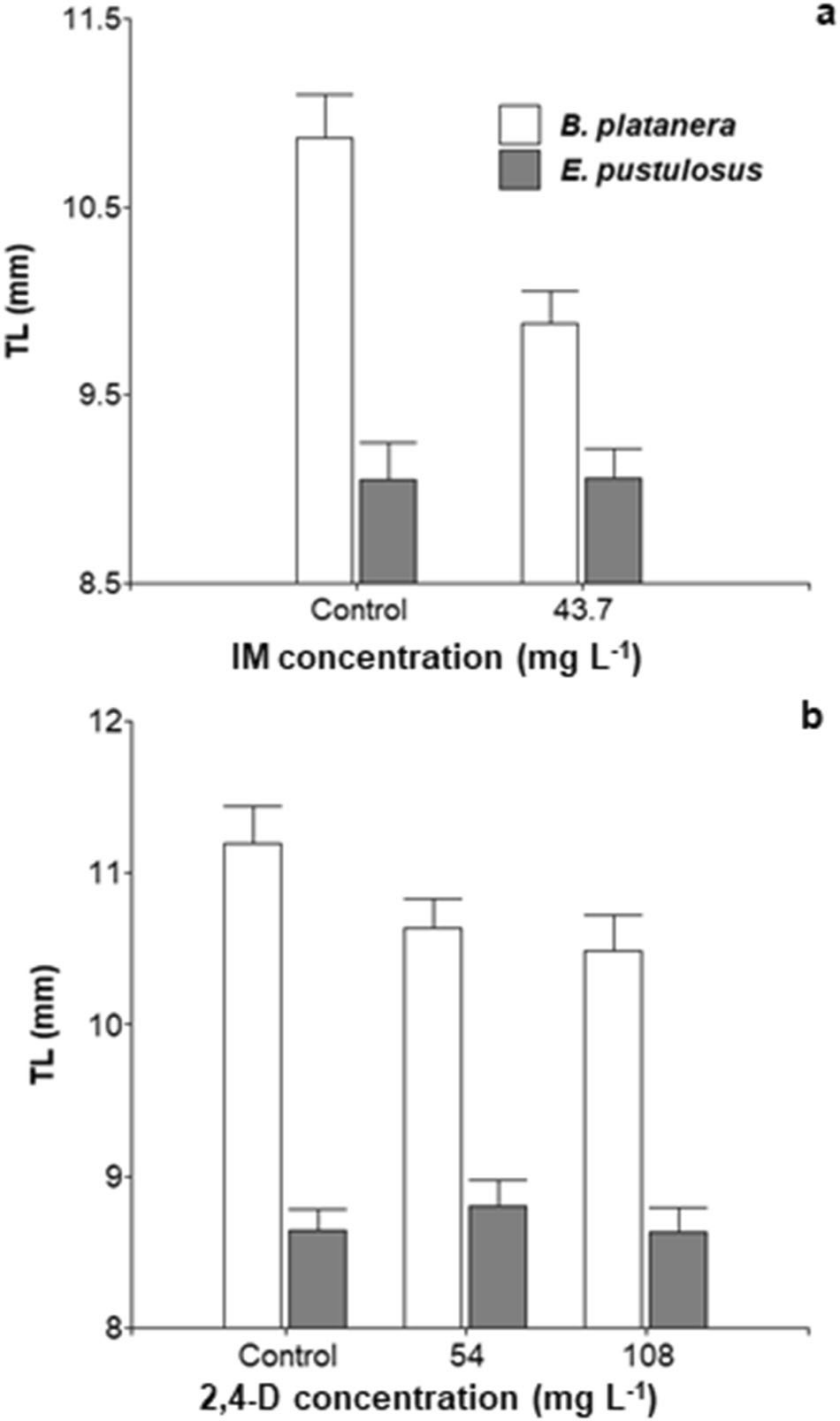
Mean total length (TL) of surviving tadpoles exposed to the pesticides IM (**a**) and 2,4-D (**b**). The bars represent the standard error

The total length (TL) of the surviving tadpoles of *B. platanera* that were exposed to the lowest experimental concentration of IM (43.7 mg L^−1^) was lower than that of the control tadpoles (*t test: t* = 3.425; *p* = 0.001, *n* = 59) (Fig. 3a). However, there was no significant difference between these concentrations in the *E. pustulosus* tadpoles (*t test*: *t* = −0.036, *p* = 0.971, *n* = 45). Sublethal concentrations of the herbicide 2,4-D (54 mg L^−1^ and 108 mg L^−1^) did not affect the total length of the tadpoles studied in either *E. pustulosus* (ANOVA: F = 0.36, *p* = 0.70, *n* = 86) or *B. platanera* (ANOVA: F = 2.76; *p* = 0.691, *n* = 89) (Fig. 3b).

Tadpoles from both species were unable to swim at the lowest sublethal concentration of IM (43.7 mg L^−1^) (100% of tadpoles were motionless). Consequently, it was not possible to perform burst swimming performance tests. Several morphological malformations, such as tail curvature and scoliosis (Aguillón-Gutiérrez 2018), were found in these tadpoles. In contrast, tadpoles of both *E. pustulosus* and *B. platanera* were able to swim at the two sublethal concentrations of the herbicide 2,4-D. Nevertheless, there were no significant differences in burst swimming performance between these concentrations and the control in the two species, *B. platanera* (ANCOVA: F = 0.582, *p* = 0.561, *n* = 89) and *E. pustulosus* (ANCOVA: F = 1.342, *p* = 0.742, *n* = 86).

## Discussion

The tadpoles of *E. pustulosus* were found to be more sensitive to IM than those of *B. platanera*. However, no significant differences (at the 95% confidence intervals) were detected between these two species regarding the herbicide 2,4-D. These findings contrast with those of Henao et al. (2022), who reported no significant differences in LC_50_ between the same two species when exposed to three organophosphorus insecticides, and Bernal et al. (2009), who found that tadpoles of *E. pustulosus* were more tolerant to the formulated herbicide glyphosate than tadpoles of *B. platanera*. It is difficult to explain the differences in acute toxicity between these species, as the same species can even respond in multiple ways to herbicides or insecticides. Changes in the commercial formulations of pesticides, the ontogenic stage of experimental tadpoles, and genetic variation among the populations studied could account for these differential responses to either herbicides or insecticides.

According to the SSD graph (Fig. 2a), B. *platanera* was the most tolerant species to the insecticide IM (LC_50_ = 211.07 mg L^−1^), while tadpoles of *E. pustulosus* exhibited intermediate tolerance. In a study conducted by Fonseca et al. (2022), *S. granulatus* tadpoles at stage 25 presented the lowest 96 h-LC_50_ values reported for IM in anurans (LC_50_ = 11.28 mg L^−1^), followed by *R. arenarum* tadpoles at stage 25 (LC_50_ = 25 mg L^−1^) and stage 27 (27 mg L^−1^). They also indicated that the LC_50_ values for these two species are well above the concentrations of IM detected in aquatic environments (mean 59.7 ng L^−1^). We have no reports of IM in surface waters near agricultural fields in Colombia. However, a meta‑analysis of neonicotinoid insecticides in global surface waters from several countries, published by Wang et al. (2023), reported a mean concentration of 119.542 ng L^−1^ for the insecticide IM. Based on this information, we also believe that our two Colombian anuran species (*E. pustulosus* and *B. platanera*) would be not at risk of lethality for this insecticide in their aquatic environments.

Regarding the herbicide 2,4-D, there is valuable information about its concentration in the environment (Islam et al. 2018). Nonetheless, there are no records for Colombia either. In Argentina, Peluso et al. (2020) reported an environmental concentration between 1 and 4 μg L^−1^ in a river in Buenos Aires Province. In Brazil, Marchesan et al. (2010) detected a concentration of 3.4 μg L^−1^ in some rivers. However, higher concentrations, up to 860 mg per hectare of 2,4-D, have been noted in aquatic environments near agricultural fields due to the runoff of agrochemicals after application (Gonzalez et al. 2016). Islam et al. (2018) also reported that the estimated environmental concentrations of 2,4-D in freshwater bodies range from 4 to 24 μg L^−1^, with levels reaching up to 4000 μg L^−1^ in agricultural fields. Taking these data together with those from the SSD graph (Fig. 2b), which indicates that tadpoles from the two species studied herein are the most sensitive to the herbicide 2,4-D, we consider that they would have a low risk of lethal toxicity to the 2,4-D herbicide, given that the LC_50_ values were notably far from those reported in the environment. Further information regarding the natural concentrations of this insecticide and herbicide in aquatic habitats in Colombia is necessary for a better understanding of the real risk of amphibian populations living in agricultural areas.

In the present study, only tadpoles of *B. platanera* showed a significant decrease in total length when exposed to sublethal concentrations of IM (Fig. 3a), but both species were unable to swim, as they remained immobile. The strong effect of IM on tadpole swimming performance parallels previous studies demonstrating that numerous insecticides impair motor activity in aquatic vertebrates, such as fishes and amphibians (Crosby et al. 2015; Triana et al. 2017; Sievers et al. 2018; Silva et al. 2021; Henao et al. 2022). This impairment is mainly attributed to the inhibitory effect of insecticides on the enzyme acetylcholinesterase, which is fundamental for vertebrate motor activity (Hoffman et al. 2003; Robles-Mendoza et al. 2009). Thus, IM may reduce an individual´s chance to escape from predators, compete for food, and survive (Kats et al. 2002; Norin & Clark 2017; Lindgren et al. 2018). In the case of 2,4-D, neither the total length nor the burst swimming performance of the tadpoles were affected. This result is unexpected because many agrochemicals are known to negatively impact growth and size during the early stages of amphibian development (Sparling et al. 2010; Baker et al. 2013; Suarez et al. 2021).

In accordance with the lethal and sublethal effects observed in the present work, IM showed greater toxicity than 2,4-D to the tadpoles of the two Colombian species studied. The differential toxicity effects between these two agrochemicals on anurans may be attributed to variations in chemical compositions and mechanisms of action between herbicides and insecticides. For instance, herbicides such as 2,4-D act as hormonal disruptors that affect normal plant growth and development, whereas insecticides such as IM act on the acetylcholine receptors of the central nervous system of insects. It is possible that IM exerts a stronger effect on anurans than 2,4-D because they also have acetylcholine receptors where IM may act; even though it is considered that IM has a low affinity for these receptors in vertebrates (Dalefield 2017). Given our results and those reported by Wrubleswski et al. (2018), who evaluated the acute and chronic toxicity of several pesticides on *Physalaemus cuvieri* tadpoles, we postulate that insecticides may generate more deleterious effects on amphibians than herbicides.

In conclusion, we found that tadpoles of *E. pustulosus* were more sensitive than those of *B. platanera* when exposed to the insecticide IM, but no differences were detected between these two species in relation to the herbicide 2,4-D. Additionally, the SSD graph shows that tadpoles from *E. pustulosus* and *B. platanera* are the most sensitive species to the herbicide 2,4-D. Furthermore, when comparing the LC_50_ values obtained in this study with data recorded in aquatic habitats from agricultural fields for both pesticides, it appears that the two species of Colombian anurans studied may face a low risk of acute lethality. However, exposure to nonlethal concentrations of the insecticide IM revealed that this pesticide can still induce deleterious effects on amphibians, potentially impacting species fitness. Therefore, toxicity tests evaluating only lethal concentrations may underestimate the real impact of agrochemicals, particularly if the lethal concentrations notably differ from the recommended spray concentrations in the field. Finally, the insecticide IM was more toxic than the herbicide 2,4-D, as it presented the highest mortality and caused a significant decrease in the total length and swimming activity of tadpoles. Consequently, we propose that insecticides may exert more pronounced toxic effects on anurans than herbicides.

## Data Availability

All data on the toxicity assays of both agrochemicals of this study are available in Figshare with the identifier 10.6084/m9.figshare.25860574.
